# Enhancement of Cutting Performance of Ceramic Tools by Addition of Exogenous Precursor Restorers

**DOI:** 10.3390/ma18245498

**Published:** 2025-12-07

**Authors:** Zhaoqiang Chen, Pengcheng Song, Chuanfa Shen, Xianglong Meng, Hui Chen, Jingjie Zhang, Mingdong Yi, Guangchun Xiao, Chonghai Xu

**Affiliations:** 1 Shandong Key Laboratory of CNC Machine Tool Functional Components, School of Mechanical Engineering, Qilu University of Technology (Shandong Academy of Sciences), Jinan 250353, China; 2 Shandong Institute of Mechanical Design and Research, Jinan 250031, China

**Keywords:** ceramic tool, precursor repair agent, crack healing, cutting performance

## Abstract

To address brittle cracks in ceramic tools, an exogenous precursor ceramic repair agent was developed and applied to Al_2_O_3_/TiC/NiMo composite ceramic tools, which were treated by a two-step heat treatment process (heating at 3 °C/min to 300 °C for 60 min, heating the sample at 5 °C/min to 500, 600, 700, 800, and 900 °C, holding each for 60 min). The crack healing mechanism and temperature dependency of the repair agent were investigated. Cutting performance, including surface roughness, cutting force, and tool life, was optimized using an L9(34) orthogonal design. The results show that at 900 °C, the repair agent decomposed to form SiOC (Silicon Oxycarbide) amorphous phase and TiB_2_ reinforced phase, filling the cracks and achieving atomic-level diffusion bonding. The flexural strength of the repaired sample recovered to 79.9% of the initial value (456.5 MPa), a 196.4% increase compared to the unrepaired sample. Optimal cutting parameters were found to be a cutting speed of 200 m/min, back draft of 0.1 mm, and feed of 0.1 mm/r. Under these conditions, surface roughness was 0.845 μm, cutting temperature was 258 °C, and stable tangential force was 70 N. The effective cutting distance of the repaired tool was increased from 1300 m to 1700 m. Wear was primarily abrasive and adhesive wear, and the SiOC phase formed by the repair agent helped to fill and repair the flank, thus extending tool life.

## 1. Introduction

Ceramic materials occupy an important position in the field of cutting tools due to their advantages such as high hardness, good wear resistance, good chemical stability, good high temperature performance and low price [1]. However, their intrinsic brittleness leads to cracking or even fracture under mechanical loading or thermal shock [2,3], which seriously restricts engineering applications. To address this limitation, researchers have adopted two methods: one is to enhance the fracture toughness of ceramics with the help of adding whiskers, particles and fibers [4,5,6,7]; the other is to promote advanced ceramics to have the ability of crack repair [6,8,9,10,11]. Although traditional toughening techniques can enhance fracture toughness, they often come at the expense of material hardness. In contrast, crack repair can be triggered by autonomous or micro-intervention repair to synchronize the restoration of performance, life extension, cost reduction, and efficient use of resources [12,13,14,15].

Research related to crack repair of ceramic tools is currently focused on chemical re-action repair, i.e., through the reaction of the second-phase material with air or other sub-stances, and the use of its products to achieve the filling and repair of cracks [16,17]. Chen et al. [18] investigated the effect of heat treatment temperature and time on the crack healing behavior of Al_2_O_3_/TiC/TiB_2_ ceramic cutting tool materials and the recovery of material flexural strength. The results show that the cracks on the surface of the material can be completely filled and healed by the oxidation products, in which the flexural strength of the specimens containing prefabricated cracks recovered to 91.35% of that of the smooth specimens after heat treatment at 700 °C for 60 min. Li et al. [19] prepared Al_2_O_3_/TiB_2_/TiSi_2_ ceramic materials by vacuum hot pressing and studied their crack healing behavior by high temperature oxidation treatment. The results showed that the flexural strength of the specimens with 150 μm and 300 μm cracks was completely recovered after treatment at 800 °C for 90 min, and even reached 107.25% of that of the smooth specimens. Zhang et al. [20] prepared Al_2_O_3_/TiC/TiB_2_/h-BN@Al_2_O_3_ ceramic tool materials. After air heat treatment at 700 °C for 60 min on 400 μm cracked specimens prefabricated on ceramic tool materials, the cracks were completely healed, and the flexural strength was restored to 98.2% of that of the smooth specimens. In the above experiments, the cracks are repaired by the oxidation products generated by the high temperature reaction between TiB_2_ and the O element in the air, which belongs to the repair of ceramic material itself (non-exogenous), but the repair effect is easily limited by the size of the cracks, and the repair process will change the structure of the tool material so that the performance is not enough to recover, and the compatibility with the tool substrate is poor and easy to introduce impurities to affect the performance of the stability of the tool.

The ceramic precursor material, on the other hand, is able to realize the transition from organic to inorganic with the increase in temperature, and has excellent high temperature resistance and mechanical properties. In particular, materials such as siloxanes are potential restorers of ceramic materials because of their excellent fluidity and repair agent ability. Based on the special properties of precursor ceramics, they have been used as high-temperature-resistant adhesives for ceramic materials by researchers [21,22], but the crosslinking pyrolysis process always involves the detachment of small molecule materials, which leads to low ceramic yields and defects such as porosity. In this regard, an appropriate amount of ceramic filler can be added to the repair agent to inhibit volume shrinkage and pore defects, and improve the mechanical strength and sintering yield [21,22]. Chen et al. [23] prepared a modified ceramic precursor high-temperature-resistant adhesive for joining zirconia ceramics and titanium alloys, with a bond strength of about 20 MPa after sintering at 1000 °C and exhibiting good oxidation resistance.

Based on this, in this study, an exogenous precursor ceramic repair agent was proposed using Al_2_O_3_/TiC/NiMo composite ceramic tool as a repair matrix. Combined with Al_2_O_3_/TiC/NiMo composite ceramic tools, the aim is to systematically investigate the repairing effect of this repair agent on ceramic tool cracks under different high temperature treatment conditions, especially the degree of recovery of the key mechanical properties of ceramic tool materials, and investigate the effect of temperature variation on the repair performance. Meanwhile, this study will evaluate the performance of the repaired tool in the actual cutting of 304 stainless steel, and optimize the cutting parameters through orthogonal experimental design, with surface roughness, cutting force and tool life as the core evaluation indexes. It will also investigate the effectiveness of this repair method to enhance the comprehensive service performance of ceramic tools, and provide an effective exogenous repair strategy to solve the problem of life shortening caused by brittle cracks in ceramic tools.

## 2. Materials and Methods

### 2.1. Experimental Raw Materials and Preparation

The substrates of the repair materials were selected from polymethylhydrosiloxane (PMHS, average molecular weight 1700–3200, purity 98.5%, hydrogen content 1.6%, Shanghai McLean Biochemical Technology Co., Ltd., Shanghai, China) and tetramethyltetravinylcyclotetrasiloxane (D^4^Vi, density 0.997 g/mL, purity 95%, Shanghai McLean Biochemical Technology Co., Ltd.) The filler was selected as nano TiB_2_ powder (99.9% purity, 40 nm, Shanghai Chaowei Nano Technology Co., Ltd., Shanghai, China) The ceramic tool uses Al_2_O_3_ as the matrix material, TiC, Ni and Mo as the reinforcing phase materials, and Y_2_O_3_ as the sintering additive (Shanghai Chaowei Nano Technology Co., Ltd.), and the key parameters of the material part are shown in Table 1.

**Figure 1:**
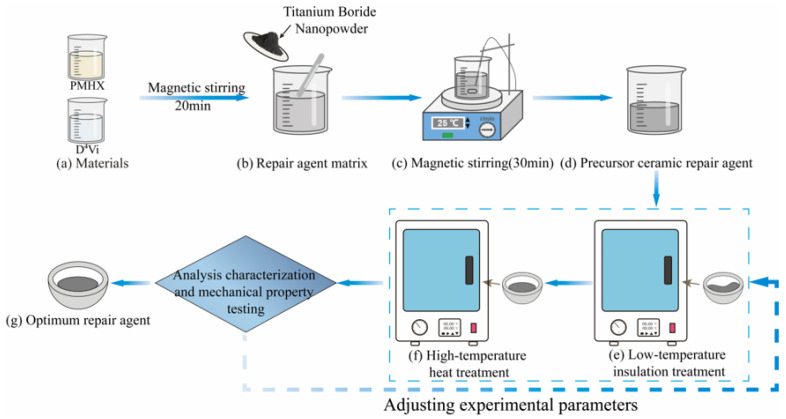
Process flow for the preparation of high temperature-resistant repair agent.

In order to investigate the optimal reaction conditions of the repair agent and the effect of TiB_2_ nanoparticles on the yield and mechanical properties of the repair agent matrix ceramics, different repair agents were prepared and analyzed. The experimental procedure is shown in Figure 1, and the specific steps are as follows: the PMHS and D^4^Vi solutions were weighed according to a specific mass ratio, and magnetically stirred at 25 °C for 20 min to promote the full mixing of the two-phase solutions, so as to obtain the matrix portion of the precursor ceramic repair agent. Based on this precursor repair agent matrix, a certain mass of nano TiB_2_ filler was weighed and added to the matrix solution, and magnetic stirring was continued for 30 min at 25 °C environment to ensure that the polymer repair material and the filler were completely mixed homogeneously, and then the precursor ceramic repair agent was produced.

To characterize parameters such as ceramic yield, the prepared repair agent was placed in a ceramic dish and heat treated using a two-step holding method. In the first step, heat the temperature to 250–350 °C at a rate of 3 °C/min under an air environment and maintain this temperature for 60 min. The second step is to put the fully cured material into the muffle furnace (SRJX-4-13, Yan Guang Instrument Co., Ltd., Shaoxing, China), heat the temperature to 900 °C at a rate of 5 °C/min, and maintain this temperature for 60 min to fully pyrolyze it to obtain the ceramic repair agent material.

### 2.2. Crack Repair Experiment

**Figure 2:**
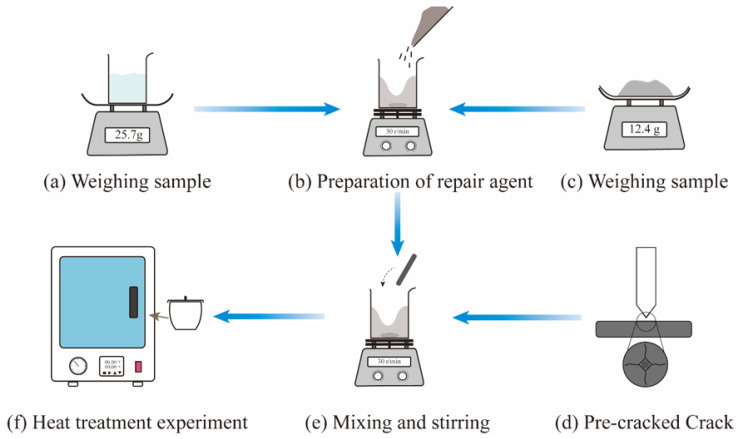
Flow of restoration experiment.

In the performance test of crack repair of ceramic materials, flexural strength can reflect the crack repair effect more significantly compared to hardness and fracture toughness. Al_2_O_3_/TiC/4 vol.% NiMo ceramic material was selected for this repair experiment. First, an appropriate amount of polyethylene glycol (PEG) dispersant was added to a beaker containing 40 mL of anhydrous ethanol, heated in a water bath, and mechanically stirred for 20 min to obtain a mixed solution of PEG and anhydrous ethanol. Then, Al_2_O_3_ (7.6 g), TiC (4 g), Ni (0.6 g), and Mo (0.3 g) powders were weighed and dispersed by ultrasonic dispersion for 30 min with the addition of anhydrous ethanol and the above mentioned mixture. Ball milling on the resulting multiphase suspension was performed for 48 h at a speed of 270 rad/min under a nitrogen atmosphere. After drying and sieving (200 mesh), the powder mixture was sintered by SPS (Dr. SINTER SPS-625hf, Tokyo, Japan) in a 30 mm graphite mold lined with graphite foil. The fired ceramic block was processed into a standard specimen of 3 mm × 4 mm × 25 mm, and its surface was ground and polished to a roughness of less than 0.1 μm. Then, a pre-crack is introduced at the center of the 4 mm × 25 mm surface, followed by the application of a 196 N pressure, which is maintained for 15 s. Prefabricated crack widths are greater than 1 μm, and the 40 nm filler particle size in the repair agent ensures that it enters the cracked area. Before the repair experiment, the repair agent was prepared according to the components of the repair agent with optimal repair effect. During the experiment, the ceramic specimen with prefabricated cracks was put into the repair agent and stirred thoroughly for 30 min to ensure that the repair agent fully entered the cracks. Figure 2 illustrates the process of preparing the repair agent and introducing it into the pre-crack, while Table 2 presents the heating schedule.

The specimen is ground and polished after the repair is completed, removing the repair agent adhering to the surface without destroying the original polished surface. Finally, using Shimadzu’s AGS-X5KN model testing machine (Kyoto, Japan), using the three-point bending method, the cracked surface is placed at the bottom, and the flexural strength of the specimen is tested at a loading rate of 0.5 mm/min and a span of 20 mm, and the flexural strength can be obtained by using the flexural strength formula (Equation (1)).

(1) σf=3PL2bh2

In the equation, *P* is the maximum load at the fracture of the specimen (N), *L* is the span between the support points (mm), and *b* and *h* represent the width and height of the ceramic specimen, respectively (mm).

### 2.3. Cutting Experiment

For the cutting experiment, 304 austenitic stainless steel was selected as the cutting workpiece material. The hardness of the workpiece material ranges from 15 to 30 HRC and Table 3 demonstrates the constituent elements of the workpiece and the content of each element.

The cutting tool was selected as a homemade Al_2_O_3_/TiC/4 vol.% NiMo tool with dimensions of 13 mm × 13 mm × 5 mm and tip arc γε radius of 0.2 mm, and its main mechanical properties are shown in Table 4. The toolholder selected for cutting is a CSDNN2525M1204 turning toolholder with the following geometry: front angle γo = −5°, clearance angle αo = 5°, and main deflection angle kr = 45°.

**Figure 3:**
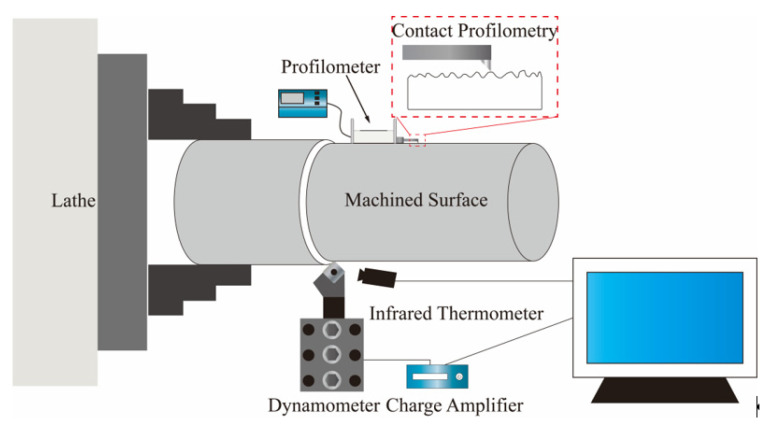
Cutting device.

Dry cutting experiments were conducted on a CDE6140A CNC lathe (Dalian, China), with real-time acquisition of three-dimensional dynamic cutting force components using a Swiss Kistler 9265A piezoelectric force measurement system (range: 1000 N) (Kistler, Shanghai, China). Simultaneously, a U.S. FLIR A320 infrared thermal imaging camera (temperature range: 0–2000 °C) (FLIR, Middletown, NY, USA) was used to monitor the temperature field distribution at the cutting interface. Figure 3 illustrates the schematic diagram of the cutting apparatus. After completing the cutting process, the workpiece surface roughness Ra value was measured on the machined surface using Beijing Times TR240 contact profilometer (Beijing TIME High Technology Ltd., Beijing, China).

The width of the wear land (VB value) on the tool’s flank was monitored every 200 m of cutting travel using the Keyence VHX-5000 3D microscope system (Shanghai, China). Tool failure was determined when VB > 0.3 mm. The tool life evaluation experiment was set up with two groups: the control group and the repair group. In the control group, the tool was used for cutting until failure. In the experimental group, the tool was repaired when the VB threshold reached 0.25 mm. The worn tool was immersed in a repair solution and placed in a muffle furnace for heat treatment. Afterward, the repair layer was removed by precision grinding and polishing to ensure no damage to the substrate and no residual material on the surface. The repaired tool was then put back into cutting until tool failure. All experimental samples were finally analyzed for wear morphology and elements by scanning electron microscopy–energy dispersive spectroscopy (SEM-EDS).

By reviewing the process requirements for machining 304 stainless steel with ceramic tools in the literature [24,25], the experimental parameters were optimized and adjusted as follows: the speed interval was maintained at 100–300 m/min, the amount of back-eating was reduced to 0.1–0.3 mm, and the feed was adjusted to 0.1–0.2 mm/r (the distance the tool moves relative to the workpiece during one complete revolution). In order to systematically evaluate the interaction effect of cutting amount on machining quality, nine groups of process combinations were designed based on the L9(34) orthogonal test table, and the influence weights of the three parameters of cutting speed (vc), back-eating amount (ap), and feed (f) were ranked by the analysis of polarity, using the surface roughness Ra as the evaluation index. The factor levels are shown in Table 5.

## 3. Results

### 3.1. Crack Repair Performance

In the repair process of ceramic materials, the temperature has the most significant effect on the repair effect [26,27]. In order to investigate the influence of different temperatures on surface crack repair, this study observes the crack morphology under different repair temperatures by SEM.

**Figure 4:**
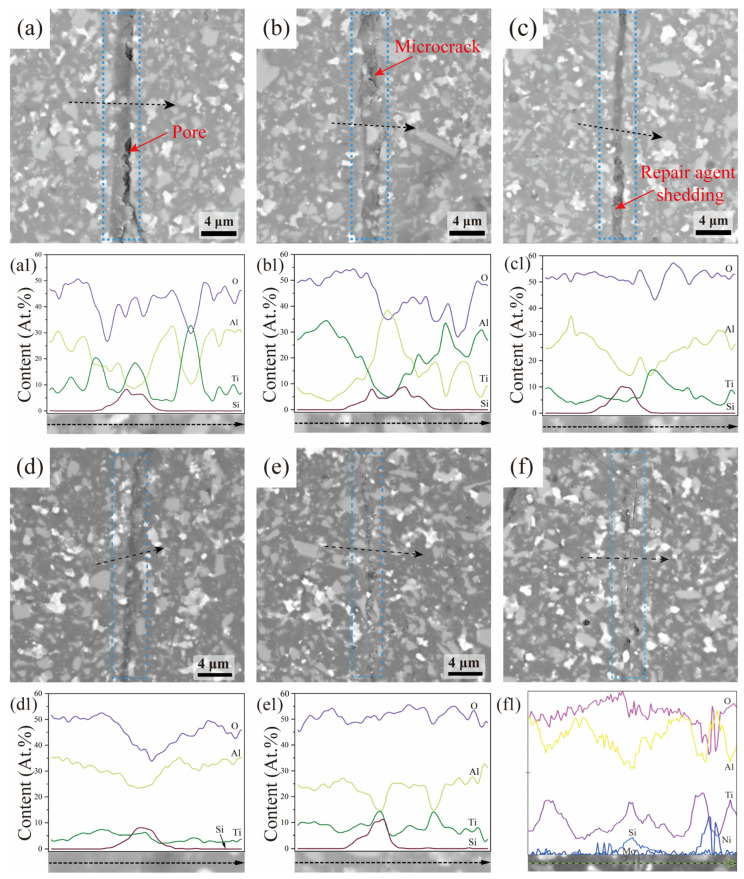
Crack repair morphology and EDS spectrum analysis at different temperatures: (**a**,**a1**) 500 °C, (**b**,**b1**) 600 °C, (**c**,**c1**) 700 °C, (**d**,**d1**) 800 °C, (**e**,**e1**) 900 °C, and (**f**,**f1**) 1000 °C.

As shown in Figure 4a–e, the crack repair effect shows a significant temperature-dependent evolution pattern. Under the 500 °C treatment condition, the repair material shows good crack filling ability, it can effectively fill the main damage area, and the surface morphology is flat without obvious signs of shrinkage. However, micrometer-scale gaps still existed at the interface between the healing material and the substrate, indicating that the interfacial bonding properties of the two still need to be improved. The energy spectrum analysis shows that Si and Ti elements are mainly distributed in the crack region, which is consistent with the composition of SiOC amorphous phase and TiB_2_ reinforced phase generated by pyrolysis of the healing material, confirming that the crack repair is mainly based on the physical filling as the main mechanism. When the temperature was raised to 600 °C, the interfacial bonding strength did not achieve substantial breakthrough, and although no large-area peeling phenomenon occurred, micron-level gaps still existed, indicating that the degree of material densification and interfacial connectivity still need to be optimized. After the temperature rises to 700 °C, the interface forms a continuous bonding region, but there is a local phenomenon of peeling off of the repair layer, and the residual cracks are still clearly visible. This is attributed to the fact that the dynamic equilibrium between the shrinkage stress [28] and the interfacial bonding of the healing material is broken at high temperatures. Further heating to 800 °C resulted in a seamless bonding between the healing material and the substrate, with a significant improvement in surface smoothness. However, due to the influence of thermal shrinkage stress, some areas still exhibit traces of cracks. When the temperature reaches 900 °C, the cracks are completely filled and there are no visible defects on the surface. The high temperature promotes the diffusion of the material, so that the edges of the residual cracks show diffuse characteristics. Energy spectrum analysis confirms that the distribution of elements in the healing zone is consistent with the original material, confirming the dominant role of the healing material. The repair condition at 1000 °C is shown in the figure. At this temperature, there was no significant improvement in crack repair, and the generated SiOC phase would transform into SiC_4_ and SiO_4_ at high temperatures. Combined with the upper limit of temperature control of the equipment, 900 °C was determined to be the optimal repair parameter, at which the technical requirements for structural repair were met.

**Figure 5:**
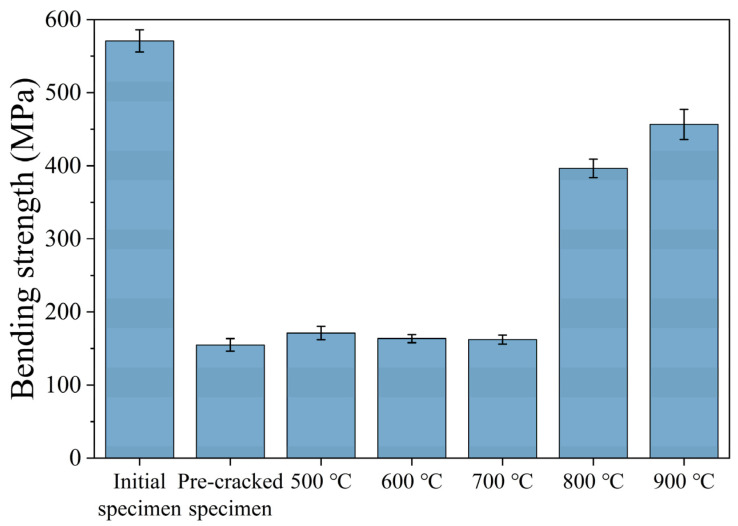
Flexural strength under different repair conditions.

This study systematically evaluates the effect of temperature on the repair effect by comparing the evolution of the flexural strength of ceramic specimens at different repair temperatures. The data in Figure 5 show that the original specimen flexural strength was 570.9 MPa, and the strength of the cracked specimen plummeted to 154 MPa (only 26.9% of the original value). In the 500–700 °C repair interval, the flexural strength of the repaired specimens increased only slightly to 166 MPa (about 29% of the original value), indicating that the repair effect at this stage was weak.

The analysis shows that the main reasons for the limited effect of low and medium temperature repair are insufficient pyrolysis reaction of the repair agent, which exists on the surface of the substrate only in the form of physical attachment; insufficient strength of the repair material itself; and the lack of an effective chemical bonding interface. The combination of all three results in the repair agent had only a limited filling role.

When the temperature was increased to 800 °C, the strength of the specimen was significantly recovered to 396 MPa (up to 69.3% of the original value); it was further increased to 456.5 MPa at 900 °C (recovery rate of 79.9%). The performance enhancement comes from the dual optimization under high temperature environment: firstly, the repair agent is fully pyrolyzed to form a dense amorphous ceramic phase, which realizes the effective filling of cracks, and the mechanical properties of the amorphous ceramic phase are relatively better, which effectively prevents the crack extension; secondly, the high temperature promotes the diffusion of interfacial atoms, which forms a stable chemical bonding, and strengthens the load-bearing capacity of repair area. Microanalysis confirms that this composite repair mechanism can effectively inhibit crack extension and realize systematic recovery of mechanical properties.

### 3.2. Cutting Repair Performance

#### 3.2.1. Improvement in Cutting Performance

The obtained surface roughness values were filled in the orthogonal test table and the analyzed results are shown in Table 6.

From an intuitive analysis of Table 6, it can be seen that the order of the range R of the surface roughness in the cutting experiment is RC > RA > RB. In the analysis of the orthogonal test, the larger the range, the greater the influence of this factor on the test. Therefore, the order of influence of the factors is feed rate > cutting speed > depth of cut. In addition, the mean values under different factors can reflect the optimal parameters of this factor, and the minimum value is K2 in column A, K1 in column B, and K1 in column C. Therefore, it is concluded that the optimal parameters of the cutting experiment are A2B1C1, i.e., the cutting speed is 200 m/min, the back-eating amount is 0.1 mm, and the feed amount is 0.1 mm/r.

**Figure 6:**
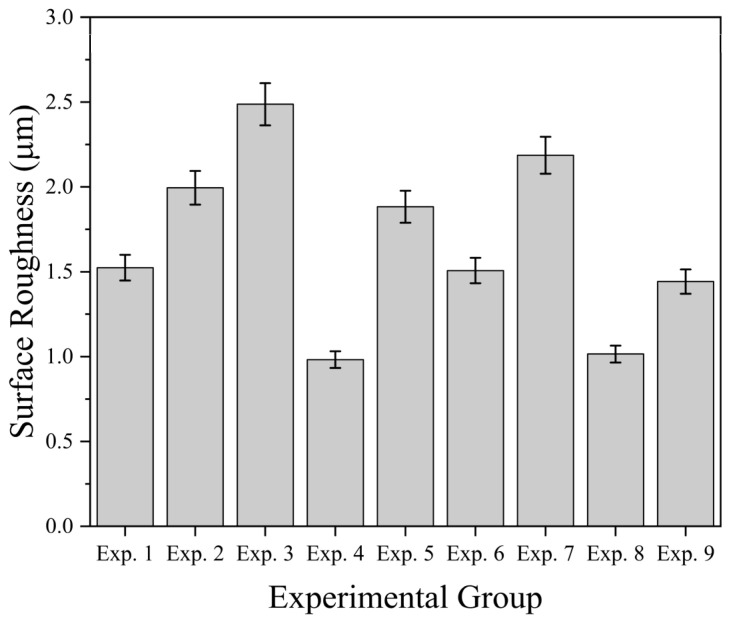
Surface roughness of workpiece.

Figure 6 shows the surface roughness corresponding to the nine sets of cutting experiments shown in Table 6.

To further analyze the impact of various factors on the results and minimize the influence of errors, an analysis of variance was conducted on the data. In Table 7, S is the sum of squared deviations (i.e., mean square) and “a” represents the mean square of the error, which was chosen to be 0.1 for this study. After calculating the mean square, if the value for a factor is less than or equal to that of the error (i.e., S ≤ a), they are categorized as errors constituting a new error. It can be seen that the sum of the squares of the deviations for factor B is less than the mean square of the error, requiring factor B to be categorized as an error and forming a new error.

In the variance calculation process, when the F-ratio is greater than the F-critical value, it indicates that the factor has a significant impact on the experiment; otherwise, it does not. Generally speaking, the greater the gap between the F-ratio and the F-critical value, the more significant the influence of this factor on the test results, or the more important this factor is [29]. According to the variance analysis results in Table 7, the F-ratio for factor C (feed rate) exceeds the critical value, while the F-ratios for factors A (cutting speed) and B (depth of cut) are both lower than the critical value. This indicates that the feed rate has a significant effect on the surface roughness of the workpiece, while the effects of cutting speed and depth of cut are not significant. This may be due to the limited variation range of cutting speed and depth of cut in the experimental design, which prevented their effects on the surface quality from fully manifesting.

**Figure 7:**
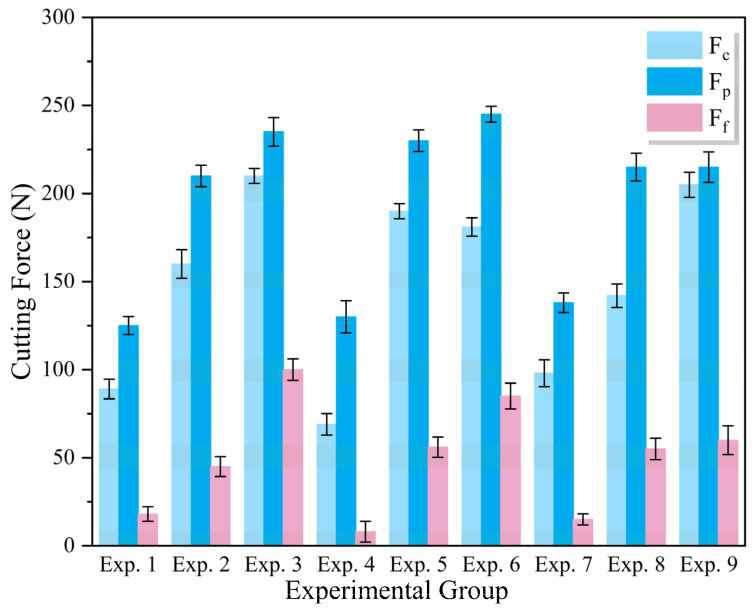
Cutting Forces under Different Cutting Parameters.

Cutting force is a key factor in the cutting process, directly affecting the material deformation and removal process, as well as influencing the surface quality of the workpiece and the tool life [30]. In the cutting process described in this paper, three types of cutting forces can be measured: tangential force, $F_{c}$, radial force, $F_{p}$, and axial force, $F_{f}$. The experimental data in Figure 7 show that the cutting force values for all conditions are concentrated within the range of 0–250 N, with the three force components following a stable ranking order: $F_{p}$ > $F_{c}$ > $F_{f}$.

**Figure 8:**
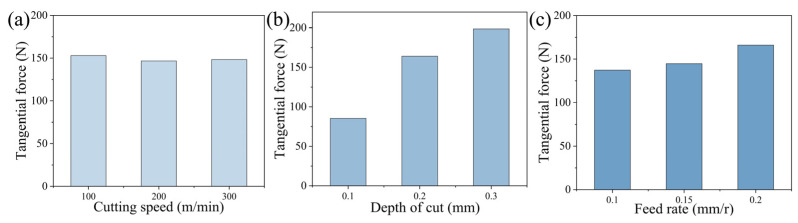
Variation trend of tangential force with cutting parameters: (**a**) cutting speed, (**b**) depth of cut, and (**c**) feed rate.

As the main cutting force, $F_{c}$ intuitively reflects the relationship between cutting force and cutting parameters, thus it was used as the evaluation index for analysis. The analysis in Figure 8 with $F_{c}$ as the evaluation index shows that during the process of increasing the cutting speed, the tangential force remains stable. The increase in the depth of cut and feed rate raises the tangential force value, among which the change in the depth of cut is particularly sensitive. This mechanical response is due to the expansion of the cutting area and the intensification of cutting deformation caused by adjustments in the process parameters, ultimately leading to an increase in the magnitude of the cutting force.

According to the analysis of the previous orthogonal experiments, the optimal parameters for cutting 304 stainless steel with ceramic tools are cutting speed of 200 m/min, back draft of 0.1 mm, and feed of 0.1 mm/r. This group of parameters was not experimentally verified in the existing experimental setups, and therefore additional tests need to be conducted.

**Figure 9:**
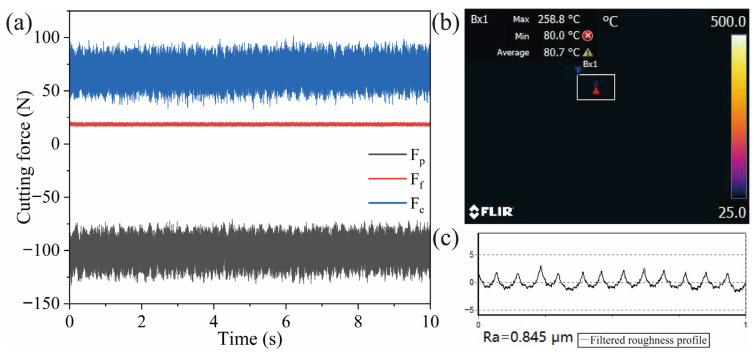
Process parameters under optimum cutting parameters: (**a**) cutting force, (**b**) cutting temperature, and (**c**) surface roughness.

Figure 9 shows the cutting force, cutting temperature and surface roughness data under the optimal cutting parameters, and Figure 9a shows the three-way cutting force in the stable cutting process. It can be seen that the cutting force under the optimal parameters is relatively lower than that under other parameters, and the cutting process is more stable, with the tangential force $F_{c}$ stabilized near 70 N, the radial force $F_{p}$ stabilized near −100 N, and the radial force is relatively small, about 18 N. At the same time, the cutting temperature of the tool is also relatively low under this cutting condition, the temperature is only 258 °C during stable cutting, the surface roughness is only 0.845 μm, and the waveform is stable, indicating that the cutting process is more stable.

**Figure 10:**
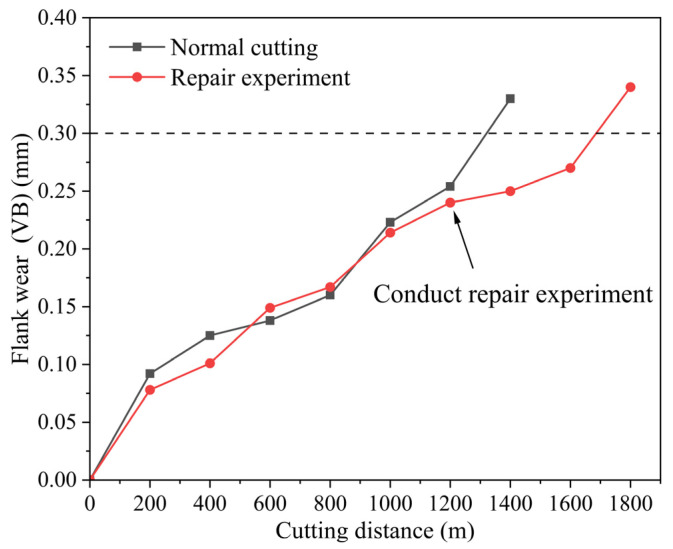
Cutting Tool Life of Ceramic Tools under Different Conditions.

In tool life assessment, the wear of the tool flank is an important indicator for evaluating tool life. During tool use, friction causes wear on the tool surface, leading to changes in the tool’s dimensions and shape. When the wear reaches a certain level, it results in a decline in the cutting performance of the tool, thereby reducing the surface quality and efficiency of the machining process. Therefore, this study uses a flank wear of VB = 0.3 mm as the criterion for tool failure. Figure 10 shows a comparison of cutting tool life experiments. It can be observed that, during normal cutting, the tool reaches a flank wear of 0.3 mm after a cutting distance of approximately 1300 m, indicating the end of the tool’s service life. In the repair experiment, when the flank wear reaches around 0.25 mm, the tool undergoes heat treatment repair. The results show that the repair experiment slows down the wear on the tool flank and extends its service life. Compared to the normal cutting process, the repair experiment can extend the cutting distance by about 400 m, with the maximum cutting distance reaching approximately 1700 m.

#### 3.2.2. Surface Morphology Analysis

To further analyze the wear patterns during the tool cutting process and compare the surface differences before and after repair, surface morphology and elemental analysis were performed on both the front and rear faces of the tool.

**Figure 11:**
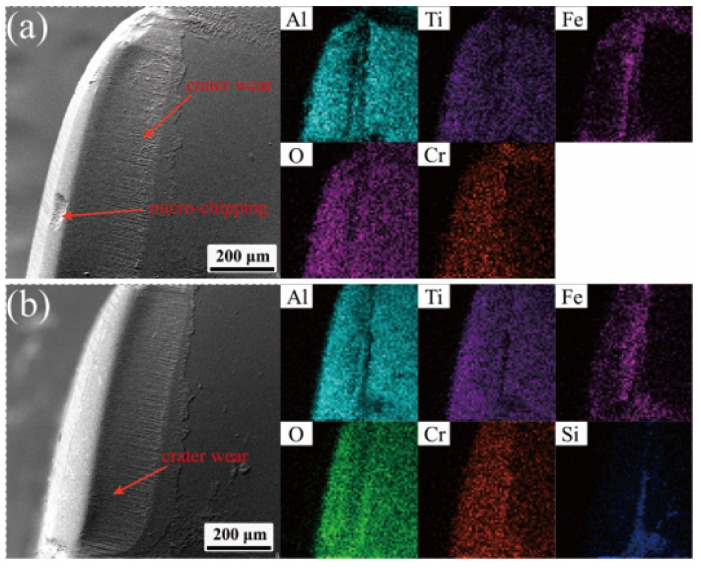
Wear and element distribution of the rake face: (**a**) normal cutting tool, and (**b**) repaired tool.

As can be seen in Figure 11, the rake face of the ceramic tool showed a narrow stripping area, forming an irregular crater wear area, while there was impact-induced chipping at the tip position. From its elemental distribution, it can be seen that there are Fe element and Cr element adhered to the tip and crater position, indicating the existence of bonding wear on the rake face, while there are obvious particle scratches on the crater, which is the main manifestation of abrasive wear. Compared to the element distribution in normal cutting, the repaired craters had the presence of the element Si which had not yet been worn away, and which was mainly derived from the repair agent.

**Figure 12:**
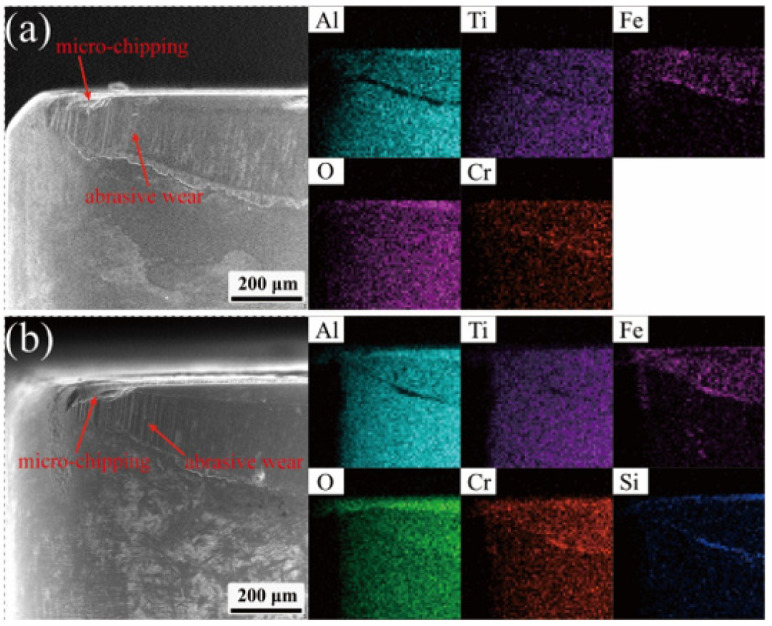
Wear and element distribution of the flank: (**a**) normal cutting tool, and (**b**) repaired tool.

Figure 12 shows the wear morphology and element distribution of the flank of normal cutting and post-repaired cutting, from which it can be seen that the flank of the ceramic tool has obvious groove scratches, the reason is that the hard particles in the workpiece in the cutting process in the surface of the tool led to scratching. And it can be seen from its elemental distribution map, the tool flank wear area has a large number of Fe, Cr elements of aggregation. This is due to the cutting process. The cutting position at the high temperature and high-pressure environment leads to the workpiece and the tool bonding to each other, which is one of the manifestations of the tool-bonded wear. In addition, there was significant chipping at the tip position, which was caused by the impact on the tool during the cutting process. Comparison of the elemental distribution of the rear flank before and after repair shows that there is an obvious aggregation of Si elements around the wear area of the rear flank after repair, because the cutting process after repair wears out the repair agent on the rear flank, so that the elements of the repair agent are only distributed around the wear area of the rear flank.

**Figure 13:**
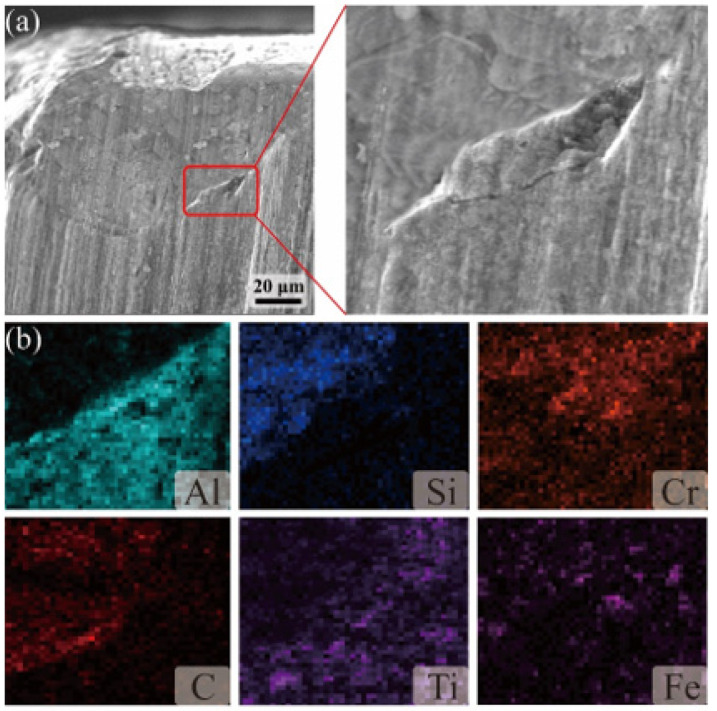
(**a**) Surface morphology of the flank after repair and (**b**) EDS spectrum analysis.

As shown in Figure 13a, after the repair experiment, the repair agent can fill and repair the position of the chipped edge of the flank, restore the original tool morphology, and replace the ceramic tool for cutting, so as to extend the cutting life of the tool; as can be seen from its local magnification, the repair agent is able to slow down the expansion of the crack, and realize the repair of the crack. Combined with Figure 13b, it can be seen that the filler elements at the chipped edge location are mainly Si, C and Ti elements, and the wear forms after repair are mainly abrasive and bonded wear.

### 3.3. Repair Mechanisms

**Figure 14:**
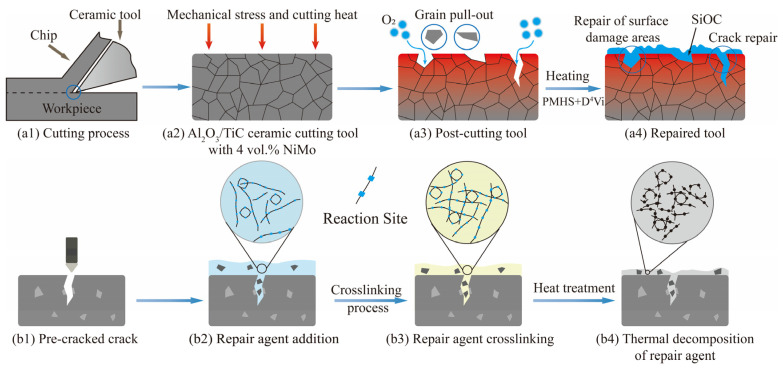
Mechanism of restorative action of ceramic materials: (**a1**–**a4**) cutting-induced repair mechanism, and (**b1**–**b4**) crack repair mechanism.

Through the analysis above, in the crack repair process of Al_2_O_3_/TiC/NiMo ceramic cutting tool materials, the repair agent is first coated on the surface of ceramic materials with cracks, and due to the good wettability of the repair agent, it is possible to ensure that the repair agent material enters into the cracked crevices. During the heat treatment process, the Si-H bond in PMHS will have a silica–hydrogen addition reaction with the C=C bond in D^4^Vi to form a three-dimensional network structure, and the macromolecules after the addition reaction cannot be dissolved in the liquid, so the curing phenomenon will be produced; due to the reordering of molecular structure and the overflow of small molecules during the curing process, there will be a certain volume contraction of the cured repair agent. As shown in Figure 14, during pyrolysis, the cured macromolecules will fracture at high temperatures to form silica–oxycarbon amorphous ceramics, realizing the transition from organic to inorganic, which is accompanied by a large number of small molecules overflowing and Si atoms oxidizing, and thus there will be a significant mass reduction and volume contraction. Meanwhile, at high temperature, the diffusion of ceramic materials will reduce the crack width, which is conducive to improving the mechanical properties of repaired ceramic materials. TiB_2_ filler, as an additive phase, can effectively improve the crack repair mode of the repair agent and enhance the repair performance. During the cutting experiment, the wear of the flanks of the tool front and rear will gradually increase with the growth of the cutting distance, which in turn affects the machining quality and tool life.

## 4. Conclusions

In this paper, the prepared exogenous precursor ceramic repair agent was applied to the crack repair experiments of ceramic tool materials, and the effects of different repair temperatures on the repair performance of ceramic tool materials were investigated. In addition, the optimal cutting parameters of 304 stainless steel as the workpiece material were investigated, while the effect of repair agent on the cutting life of ceramic tools was comparatively studied and the repair mechanism of the repair agent was analyzed.

(1) By adjusting the pyrolysis temperature of the repair agent, the repair ability of the exogenous precursor ceramic repair agent on the ceramic cutting tool material was grasped. When the pyrolysis temperature was lower than 700 °C, the repair agent basically did not take effect, and the repaired flexural strength at 800 °C could reach 396 MPa, which was restored to 75.4% of the initial specimen, and the flexural strength at 900 °C could reach 456.5 MPa, which was restored to 79.9% of the initial specimen.

(2) The crack repair morphology shows that there are obvious pores at the cracks after low-temperature repair, and the combination between the repair agent and the ceramic material is not tight; with the increase in pyrolysis temperature, the crack traces are gradually blurred and the crack position is completely filled, and the atomic diffusion at high temperature is the main reason for the blurring of the crack traces. The results of elemental analysis showed that the main substances at the cracks were the pyrolysis products of the repair agent.

(3) Through the orthogonal experiment, the optimal cutting parameters for the Al_2_O_3_/TiC/NiMo ceramic tool were determined as a cutting speed of 200 m/min, depth of cut of 0.1 mm, and feed rate of 0.1 mm/r. Under these conditions, the cutting performance is optimal, with a tangential force $F_{c}$ of only 70 N, a cutting temperature of 258 °C, a surface roughness of 0.845 μm, and a stable cutting process.

(4) A comparative experiment on tool life before and after repair was conducted. It was found that the cutting distance under normal conditions was 1300 m, while a single repair extended the cutting distance by approximately 400 m. The repair agent can effectively slow down tool wear to a certain extent and prolong tool life. The dominant wear mechanisms of the ceramic tool are abrasive wear and adhesive wear, and traces of the repair agent can be clearly observed in the elemental distribution on both the front and rear faces of the tool.

## Figures and Tables

**Table 1:** Experimental materials.

Material	Particle Size (μm)	Purity (%)	Density (g/cm^3^)
Al_2_O_3_	0.5	99.9	3.97
TiC	0.5	99.9	4.93
Ni	1	99.9	6.97
Mo	1	99.9	10.28
Y_2_O_3_	0.5	99.9	5.01

**Table 2:** Rising temperature process.

Step	Heating Rate (°C/min)	Target Temperature (°C)	Holding Time (min)
1	3	300	60
2	5	500	60
3	5	600	60
4	5	700	60
5	5	800	60

**Table 3:** Cutting workpiece element content.

Element Type	Fe	Cr	Ni	C	Mn	Si	Ps
Content (wt%)	Bal.	17–19	8–10.5	≤0.08	≤2	1	≤0.04

**Table 4:** Mechanical properties of Al_2_O_3_/TiC/4 vol.% NiMo tools.

Hardness (GPa)	Fracture Toughness (MPa·m^1/2^)	Flexural Strength (MPa)	Actual Density (g/cm^3^)
17.15	5.87	570.89	4.43

**Table 5:** Orthogonal test factors and levels.

Level	Factors			
	A	B	C	D
	Cutting Speed (m/min)	Depth of Cut (mm)	Feed Rate (mm/r)	Blank
1	100	0.1	0.1	
2	200	0.2	0.15	
3	300	0.3	0.2	

**Table 6:** Table of orthogonal tests.

Test Number	A	B	C	D	Roughness (μm)
1	1	1	1	1	1.524
2	1	2	2	2	1.995
3	1	3	3	3	2.487
4	2	1	2	3	0.982
5	2	2	3	1	1.883
6	2	3	1	2	1.507
7	3	1	3	2	2.186
8	3	2	1	3	1.015
9	3	3	2	1	1.442
Mean value K_1_	2.002	1.564	1.349	1.616	
Mean value K_2_	1.457	1.631	1.473	1.896	
Mean value K_3_	1.548	1.812	2.185	1.495	
Range R	0.545	0.248	0.836	0.401	

**Table 7:** Analysis of variance.

Factor	Sum of Squares (S)	Degrees of Freedom (F)	F-Ratio	F-Critical Value
A	0.511	2	5.162	9.000
B	0.099	2	1.000	9.000
C	1.223	2	12.354	9.000
Error	0.10	2		

Note: F(a) = 9.000, “a” represents the mean square of the error.

## Data Availability

The original contributions presented in this study are included in the article. Further inquiries can be directed to the corresponding author.
